# Supplementary material for: Are carbon emissions trading and green financial instruments synergistic? -Comprehensive quantitative research based on content analysis
Source: PLoS One. 2024 Mar 7;19(3):e0298601. doi: 10.1371/journal.pone.0298601 (PMC10919601; doi:10.1371/journal.pone.0298601)
Supplement: S1 Data — (ZIP) [file pone.0298601.s001.zip › Data/地级市Z_F工作报告.zip!/╜¡╬≈/╞╝╧τ╩╨/└┤╫╘╣1⁄2╓┌║┼í╛┬φ┐╦╩2╛▌═°í┐_2016.docx]

“十二五”时期发展回顾

“十二五”时期是我市发展历史上极不平凡的五年。过去的五年，市人民政府在省委、省政府和市委的正确领导下，坚持城市转型主战略，克服了资源枯竭、产能过剩的双重压力，致力促进经济、生态、文化、社会转型，全面完成了“十二五”规划确定的各项目标任务。2015年，全市完成生产总值912.39亿元，是2010年的1.8倍;规模以上工业增加值429.2亿元，是2010年的1.9倍;财政总收入130.48亿元，是2010年的2.03倍;全社会固定资产投资1026.74亿元，是2010年的2.5倍;社会消费品零售总额303.06亿元，是2010年的1.9倍;外贸出口12.53亿美元，是2010年的2.81倍;实际利用外资3.08亿美元，是2010年的1.89倍。全市金融机构本外币各项存款余额是2010年的2.03倍，本外币各项贷款余额是2010年的2.39倍。全市银行机构达到8大类20家，是2010年的2.5倍。招大引强和产业招商成效显著，五年来共引进省外5000万元以上项目739个，引进资金1397.84亿元。

经济转型扎实有效

经济结构逐步优化，三次产业比由2010年的8.1：63.3：28.6调整为6.9：56.7：36.4。农业经济稳步发展。粮食单产连续12年居全省第一。农业现代化稳步推进，新型农业经营主体发展到4523个，比2010年增长117%。市级以上农业产业化龙头企业总数达到163家(其中国家级2家、省级55家)，比2010年增长44.5%。种养大户、家庭农场、专业合作社分别达到2004个、306个、1822个，比2010年分别增长59%、130%、202.61%。湘东区被评为全国农田水利基本建设先进县区和全国绿色食品原料标准化生产基地。芦溪县被认定为第三批国家现代农业示范区，其他县区实现了省级示范区全覆盖。杂交水稻制种、绿色生猪、油茶、油菜、花卉苗木等特色产业不断发展壮大。工业经济逐步转型。煤炭、陶瓷、水泥、烟花爆竹和冶金五大传统产业转型升级步伐加快。粉末冶金、先进装备制造、节能环保、新材料、新能源、生物医药等新兴产业迅速壮大，在全市主营业务收入中的比重达到33%，比2010年提高16个百分点。全市综合创新能力进入全省第一序列，连续4届8年获得“全国科技进步先进市”称号，被列为“国家创新型试点城市”。第三产业发展迅猛。2015年实现旅游综合收入197亿元，比2010年增长411%。文化产业稳步发展，2015年实现文化产业主营业务收入312亿元，比2010年增长202%。金融业稳步发展，全面实施“1+10”金融改革，我市成为全省唯一的全国中小企业信用体系建设试验区。全市挂牌新三板企业5家。民营经济活跃，全市民营经济工业增加值、完成税收、提供就业岗位均占全市总量的一半以上。

生态转型稳中有进

“十二五”期间，完成水利项目462个，总投资32.92亿元，其中争取上级资金21.56亿元;总投资和争取上级资金分别是“十一五”期间的2.6倍和2.3倍。完成造林绿化54.3万亩，森林抚育76万亩，低产低效林改造13.2万亩，森林覆盖率提升到66.87%，比2010年提高3.36个百分点。富裕和谐秀美乡村建设快速推进，五年累计投入资金6.43亿元，在全市1808个自然村开展新农村建设工作，成功打造了319国道、320国道、芦万武公路等5条富裕和谐秀美乡村示范带。生态文明建设成效显著，生态环境持续改善，成功创建国家级生态乡镇12个、省级生态乡镇17个、省级生态村35个。芦溪县被纳入全国重点生态功能区和全省生态文明先行示范县。总量减排成效明显，全市累计投入减排资金约14亿元，共完成减排项目约600个，全市钢铁、水泥、火电、玻璃行业的脱硫、脱硝、除尘设施全部建成并投入营运。污染防治扎实有效，空气质量有所改善。水资源管理与保护体系日臻完善，投资7.3亿元的山口岩水利枢纽工程全面竣工，同步建成了总投资达1.2亿元的白源水厂，新增城市供水能力10万吨/日，城乡供水能力大大提升。投资3.7亿元的寒山水库开工建设。水源地保护工作取得明显成效，全市水功能区水质达标率达到92%以上。

文化转型亮点纷呈

重点布局规划了一批重点文化产业园区建设，初步形成了动漫创意、印刷包装、文娱演艺、文化旅游、广播影视等五大主导产业。全市有国家级、省级文化产业示范基地分别为1家和5家，获全省“十佳乡村文化旅游示范点”称号1家。广播剧《本色》获中宣部“五个一工程奖”。话剧《老阿姨》在全国范围内成功演出62场次，萍乡采茶戏《有事找老杨》荣获“玉茗花”大奖，《寒酸的县委办，不寒酸的民生》荣获中国新闻奖一等奖。湘东皮影戏《杨宗保破阵》获全国皮影展演暨第十二届中国民间文艺山花奖银奖。《莲花打锡》列入第四批国家级非物质文化遗产代表性项目保护名单。旅游业发展迅猛，提前实现旅游人数和旅游综合收入双翻番。武功山景区进入创5A级景区预备名单，全市A级景区由2家增加到17家。武功山、安源景区产业集群进入全省重点旅游产业集群行列，芦万武公路、市旅游集散中心、杨岐山游客服务中心、孽龙洞改造等一批重大旅游项目顺利完工，五年累计投入49.16亿元。首次承办了“中国红色旅游博览会”，连续举办了武功山国际帐篷节、莲文化旅游节等国际、国内有影响的节庆活动。乡村旅游蓬勃发展，涌现出一大批国家级、省级乡村旅游示范点。全市有国家级休闲农业与乡村旅游示范点3家，中国乡村旅游模范村、模范户各1家，中国乡村旅游金牌农家乐27家，江西省3A级以上乡村旅游示范点17家。

社会转型成效明显

五年累计投入民生类资金465亿元，全面完成国务院、省政府下达我市的各项民生工程指标。社会保障水平稳步提升，截止到“十二五”末，全市城镇职工基本养老保险参保人数35.81万人，基金征缴总额54.27亿元;全市城镇基本医疗保险参保人数110.36万人，基金征缴总额36.4亿元;全市城乡居民基本养老保险参保人数60.58万人;被征地农民参加养老保险人数2.49万人。全力打造食品药品安全城，确保了群众饮食用药安全。全面推进医疗服务能力建设，居民健康水平稳步提升，人口保持均衡发展。妇女儿童发展环境进一步优化。全面实行义务教育免试就近入学，义务教育巩固率达到95.2%;普通高中阶段毛入学率达89.2%;职业教育就业率达87%;加快高等教育发展，萍乡高专成功升本。市人民医院、安源大剧院、市老年大学、市委党校和市廉政中心全面完工并启用。就业创业同步推进，全市城镇新增就业17.86万人，困难群体(4050人员)就业1.75万人;城镇登记失业率基本保持在3.5%左右;零就业家庭就业安置率100%;农村劳动力转移培训8.35万人。保障性安居工程打造了全国保障性住房建设的“萍乡样本”，得到了国务院李克强总理的充分肯定和特别批示。“十二五”期间，全市完成保障性住房建设任务17141套，完工率100%，改造城市棚户区22121户，面积188.85万平方米。补助农村危房改造31006户，争取上级补助资金3.3亿元。沪昆高铁萍乡北站投入使用，萍洪高速、昌栗高速(上栗段)顺利通车。衡茶吉铁路的贯通，结束了莲花县无铁路的历史;吉莲高速通车，实现了县县通高速、乡乡通国省道的目标。国省干线升级改造开工建设300公里，完成170公里，完成投资29.18亿元。扶贫脱贫成效显著，投入扶贫资金6.4亿元，完成建设项目1592个，完成了10455人避灾移民、深山移民和生态移民搬迁任务，解决46052人贫困人口温饱和脱贫问题。双拥创建成果丰硕，有望连续六届创建全国双拥模范城。积极实施“专业治安”和“科技兴安”战略，按照“企业可防，政府可控”安全路线图，建立并完善了一系列高危行业安全生产管理制度和安全生产监管机制，各类安全生产事故死亡人数大幅度下降，2011、2012、2013年市政府连续三年被评为“全省安全生产工作先进单位”。社会保持和谐稳定，龚全珍工作室和杨斌圣工作室遍布全市，全市公众安全感居全省前列。“六五”普法教育全面完成。法治政府和廉洁政府建设扎实推进。

2015年工作回顾

2015年,是全面深化改革的关键之年,是全面依法治国的开局之年,也是全面完成“十二五”规划的收官之年。在省委、省政府和市委的正确领导下，在市人大、市政协的监督支持下，全市主动适应经济发展新常态，坚持“奋力把经济搞上去，切实把作风正过来”的总要求，经济社会保持平稳健康发展，主要呈现以下六个特点：

经济运行平稳有序

主要经济指标实现稳步增长，全年全市生产总值增长8.9%;规模以上工业增加值增长8%;全社会固定资产投资增长13.3%;社会消费品零售总额增长10.2%;财政总收入增长11.5%;实际利用外资增长9.8%;城镇居民人均可支配收入和农村居民人均可支配收入分别增长8.9%和10%。农业经济发展较好，实现农业总产值98亿元，农业增加值63.3亿元，均增长4%。工业经济保持“稳中有增、结构趋优”的良好态势，实现主营业务收入1682.1亿元，增长3%;实现利税总额277.9亿元，增长0.4%;完成工业固定资产投资664.4亿元，增长7.1%。湘东工业陶瓷、芦溪电瓷、上栗粉末冶金获批省重点产业集群和省示范产业集群。第三产业增长较快，大力实施旅游景区双创5A战略，扎实推进旅游重大工程建设，全年接待游客2744.7万人次，增长25.3%;旅游综合收入增长37.65%。武功山温泉度假村和武功山游客服务中心基本完成。完成了金顶区块整治、安源矿山公园博物馆等旅游景区项目。安源国家矿山公园顺利通过综合评议验收，获国土资源部正式命名。安源锦绣城、荷花博览园、杨岐山景区获评为国家4A级旅游景区。文化产业有序推进，文化产业主营业务收入增长16%。各项金融指标逆势上扬，全市金融机构人民币各项存款余额790.34亿元，增长16.25%,比年初增加110.5亿元;金融机构人民币各项贷款余额539.29亿元，增长19.39%,比年初增加107.77亿元。

项目建设持续推进

全年新开工亿元以上项目119个，竣工投产亿元以上项目84个。在建1000万元以上工业项目209个，计划总投资367亿元，本年完成投资150.5亿元。全市共有重点项目106个，总投资750.73亿元，列入第一批省重点项目14个，列全省第三。招商引资成效显著，大力推进民企入赣，通过对接央企、民企和科企，自主举办专题招商会26场，新签约项目136个，签约金额416.71亿元。德博科技二期、长竣特种材料生产线等一批重点工业项目顺利竣工。华能安源电厂#1、#2机组正式投产，成为我国首座建成投产的超超临界二次再热发电厂。中节能50MWp莲花光伏电站配套110KV输电线路项目建成并网。中环南路、安源南大道(319国道)改扩建工程基本建成通车。渝长厦铁路、萍莲高速等重点项目争取均有所进展。推进了中材科技风电叶片、润达国际、赣湘国际物流港、赣湘国际汽车城、美佳华商业广场等一批项目。生活垃圾、污泥干化焚烧发电项目主体工程开工建设。

城乡建设统筹发展

成功获批国家海绵城市建设试点城市，赢得了萍乡城市建设的历史性机遇。坚持科学规划，七项专项规划和三个技术标准为海绵城市建设奠定了坚实基础。制定了《萍乡市海绵城市试点建设三年行动计划》和三年建设项目计划，海绵城市三年建设项目159项，估算总投资46亿元，目前已开工项目18个，完成投资5.5亿元。积极探索PPP模式，狠抓PPP项目平台公司建设，确定了PPP项目第三方中介机构。新型城镇化建设亮点纷呈，“多规合一”城乡规划体系建设全面启动。全年开工城镇化项目132个，完工24个，完成投资46.4亿元。房地产市场总体平稳，商品房销售情况良好，全市商品房成交面积139.48万平方米,增长62.13%;商品房成交金额64.71亿元,增长71.53%。全力抓好武功山中大道改造、安源北大道改造、玉湖公园提升等海绵城市试点建设项目;南正街旧城改造征拆工作基本完成，鳌洲书院等旧城改造项目开始启动。成功争取列为全国第二批59个水生态文明试点城市之一，已通过立项落实建设项目27个，总投资约20亿元。大力开展全国文明城市创建和城乡环境卫生综合整治行动，城乡面貌日益好转。新农村建设有序推进，大力开展村庄整治建设，积极推进人居环境综合治理，全面推动新农村建设发展升级。全市198个新农村建设点遍布43个乡镇，惠及1.38万农户、55260人。

民生事业扎实有效

全面完成2015年省政府下达我市的50项民生工程指标。民生支出达126.48亿元，占全市公共财政支出的68.4%。全年保障性住房新开工2060套，开工率100%;基本建成2991套，完成率100%。各类棚户区改造开工10040户，开工率100%。社会救助水平进一步提高。城乡低保平均保障标准分别达到470元、260元，均高出全省平均水平20元。大力开展精准扶贫工作，群众生产生活条件进一步改善。投资近5000万元新增城市供水管网37公里，改造供水管网61公里，全面实现了中心城区三个水厂所供自来水互联互通、互为备用水源。城市供水量有了充分保证，水压有了明显提高，水质有了根本改善。建成了农村饮水安全工程42处，解决了2.9万农村居民和3.7万农村师生的饮水困难。社会保障体系不断健全。完善了被征地农民养老保险政策，切实维护被征地农民合法权益。连续11年按时足额为企业退休人员上调养老金，人均月增资270.43元，增长17.1%，全市企业退休人员人均月养老金标准达1848.41元，高于全省平均水平。在全省率先出台了《萍乡市城镇居民基本医疗保险门诊统筹暂行办法》和《萍乡市城镇居民大病保险工作实施方案》，城镇居民大病保险可全面实现在定点医院即时结算，大病保险基金使用率达87.5%，城镇职工、城镇居民医保政策范围内的报销比例分别达到81.3%和72.5%。城镇居民基本医疗保险最高支付限额由9万元提高至10万元。就业创业工作成效显著，建成创业孵化基地8个，成功孵化企业280余家，带动就业1177人。全年新增城镇就业33600人，新增农村劳动力转移就业20500人。知识产权工作位居全省前列，全年专利申请量1411项，增长63.5%，列全省第二;授权量771项，增长125.4%，列全省第一。萍乡经济技术开发区被认定为全省唯一的国家知识产权试点园区。教育投入不断提高，全市财政性教育支出约25亿元。启动了安源文化博览园、安源红领巾少儿活动基地等项目建设。在全省设区市中率先实现每个县(区)都有3厅以上数字影院的目标。大力开展“普惠金融”活动，在全省率先实现基础金融服务“村村通”工程全覆盖，建成银行消费者权益保护工作站40个。全市邮政业业务量完成2.13亿元，增长29.88%。完成通自然村公路700公里，危桥改造完成15座。优先发展城市公交，农村通班车率达到98.2%。社会保持和谐稳定。平安建设、法治建设稳步推进，获全省社会治安状况评估优秀市。信访工作取得进京访、赴省访“两个大幅下降”和“七为零”的好成绩。

各项改革稳步推进

深入推进了机构改革、文化体制改革、财税体制改革、教育综合改革、事业单位分类改革、公务用车制度改革、集体林权制度配套改革、城市管理体制改革、武功山景区管理体制改革等重点领域改革。基本完成农村土地承包经营权确权登记颁证工作，确权面积达83.11万亩，进度居全省前列。国有林场改革顺利通过国家验收。市县两级全部完成不动产登记职责整合和机构设置工作。医疗卫生体制改革扎实推进，县级公立医院改革实现全覆盖，卫生服务能力和水平进一步提升。扎实推进了省政府确定的上栗县供销社综合改革试点工作。顺利完成开发区村(社区)委换届选举工作。扎实推进了“三单两制一网一评价”工作，被列为全省唯一试点设区市。完善了行政审批事项流程再造，大大缩减了办事时限，缩减办事环节21个。在全省率先推进并完成市场监管综合设置改革，实现“三局合一”。深化“三证合一”、“一照一码”改革工作，落实各项税收优惠政策，减免税收近10亿元。经营性国有资产统一监管工作取得突破，国有资产监管新体系全面构建，国有企业“三项制度”改革顺利推进，现代企业制度日趋完善。

政府自身建设成效显著

全面推进依法行政，建立政府法律顾问委员会制度，坚决消除权力设租寻租空间，坚决纠正不作为、乱作为，坚决惩处失职、渎职，切实做到法无授权不可为、法定职责必须为。不断优化发展环境，主动服务企业、协调解决具体问题。开展影响发展环境问题专项整治工作，发现和查处了11件典型案件，2015年暗访发现违反工作纪律行为人次减少68%，投诉数量下降37%。政府信息公开扎实推进。不断加强作风建设，出台《关于重申贯彻落实中央八项规定精神有关纪律的通知》等系列制度，全市“三公”经费支出下降18.94%。开展了政府重大投资项目、重大民生工程的跟踪审计，加强对领导干部的经济责任审计。认真履行党风廉政建设责任制，党风廉政建设深入推进。办理市人大代表议案、建议59件，政协提案105件，均在法定时限内办结。

十三五”时期的目标任务

全市“十三五”经济社会发展主要目标是：生产总值年均增长8%，到2020年达1350亿元;财政总收入年均增长9%，到2020年达200亿元;固定资产投资年均增长10%，到2020年达1700亿元。与此同时，综合经济实力迈上新台阶，转型升级取得突破性进展，生态文明建设水平明显提升，民生福祉明显改善，全民文明素质和社会文明程度明显提高。

发展定位：围绕提前全面建成小康社会目标，着眼于萍乡在全国、全省区域发展格局中所处的特殊位置，努力把萍乡建设成为转型升级示范城市、创新创业活力城市、赣湘合作先行城市、生态人文旅游城市、江南特色海绵城市。主要任务和措施：

坚持转型发展，推进产业结构优化升级

一是发展优势产业。着力发展已有的优势产业，培育壮大战略性新兴产业，按照“差异化、集约化”的原则，强力推进粉末冶金、先进装备制造、新能源汽车等新兴产业集群发展，形成新的产业支撑。二是提升传统产业。大力促进陶瓷、水泥、烟花爆竹、冶金、煤炭五大传统产业转型升级。鼓励传统产业加强企业技术中心建设，使其成为传统产业突破和解决关键技术的平台。三是发展以旅游为重点的现代服务业。加快推进武功山、安源、杨岐山景区建设。力争年均旅游接待人数和综合收入分别增长20%和25%以上。突出发展生产性服务业，提升发展生活性服务业，催生新兴服务业态。大力发展文化体育产业，确保文化产业增加值年均增长10%左右，文化产业增加值占GDP的比重达7%左右。充分发挥金融在现代经济中的核心作用，保障萍乡在“十三五”期间融资额增幅高于全省平均水平。

坚持创新发展，着力提高发展质量和效益

着力提升科技创新能力。全面实施创新驱动战略，构建更加适应萍乡改革发展新常态区域创新体系。促进科技金融深度融合，逐步建立企业为主体、财政为引导、社会金融资本为补充的全方位科技投融资体系。加快实施一批产业核心技术和共性技术攻关重大专项，提升主要产业及企业核心发展能力和市场竞争力，全面建成国家创新型城市。大力推进质量强市，全面提升产品质量、工程质量、环境质量和服务质量。推动大众创业万众创新。搭建创业创新平台，实施人才强市战略，推进人才发展体制改革和政策创新，加快培养一批领军人才和学术学科带头人，培育一批省级科技创新团队。

坚持协调发展，形成城乡统筹协同发展新格局

注重规划统筹，将新型城镇化建设与海绵城市、智慧城市、水生态文明城市、国家森林城市等建设深度融合，着力解决城市空气质量不高、城市拥堵、城市内涝等问题，大幅提高城市综合承载力、集聚力和竞争力。切实抓好全国文明城市、森林城市、卫生城市“三城同创”，完善城市管网体系和海绵城市各项功能。着力创新农村社会治理，全面推动新农村建设发展升级，培育新型农业经营主体，壮大骨干龙头企业，拓宽农民增收渠道。开展城际铁路项目规划研究等前期工作，力争1至2个项目进入全省城际铁路发展规划。加快推进以萍莲高速、沪昆高速复线萍乡至醴陵、中环路等为重点的公路建设，构建以“十字型”高速铁路网、“一纵三横一联”高速公路网为主干的综合交通运输体系。加快连接核心景区旅游公路建设。规划布局通用机场，力争建设1座通用机场和若干处直升机起降点。

坚持绿色发展，着力改善生态环境

大力发展绿色经济。加强耕地保护，到2020年，全市60%以上耕地完成治理和修复。发展循环农业，到2020年，基本完成农业节能减排任务目标。提升生态优势。继续实施全市性封山育林，完成山口岩水库水源地等生态公益林修复建设，使90%以上天然湿地得到有效保护。加强资源保护和林业服务体系建设，确保全市林地保有量稳定在397万亩，公益林达到142万亩，商品林达到255万亩。加强生态环境保护。实行最严格的环境保护制度，形成政府、企业、公众共治的环境治理体系。推进多污染物综合防治和环境治理，深入实施大气、水、土壤污染防治行动计划。坚持城乡环境治理并重，加大农业面源污染防治力度。落实生态保护红线，建立生态补偿机制。改革环境治理基础制度，推行排污许可制度，推进环保系统体制机制改革。

坚持开放发展，增强合作共赢新优势

顺应区域经济一体化发展新趋势，主动对接长江经济带、全面融入长株潭城市群、新宜萍城市群、长三角和珠三角，构建全方位、多层次、宽领域开放格局。协调推进赣湘开放合作试验区上升为国家发展战略。先行启动试验区湘东园区和上栗园区，构建“一区两园”的空间格局。探索建立赣湘“共同管理”体制机制，把试验区打造成长江经济带内陆开放合作的样板。统筹推进“引进来”和“走出去”，以项目拉动投资，切实引进一批技术含量高、产业关联度大、带动能力强的优强项目。

坚持共享发展，促进社会公平和谐

增强就业和社会保障能力，加快完善覆盖城乡、人人享有、保障更好的社会保障体系。完善住房保障体系建设，以建立购租并举的住房制度为方向，把公租房扩大到非户籍人口。提高教育文化体育现代化水平。重点实施一批教育项目。推进义务教育学校标准化建设，加快普惠幼儿园建设，推进普通高中优质资源扩充工程，强化中等职业学校基础能力建设，全面推进县(区)特殊教育学校建设，加快城镇新区校区建设，不断加强教师队伍和教育信息化工程建设。不断深化教育改革，全面推进素质教育。大力支持萍乡学院和高职院校转型发展。丰富人民精神文化生活，加大公共文化产品供给，创作生产一批文化精品，按照标准化、均等化要求，推进公共文化服务体系建设。组织实施一批文化工程，保护好、利用好、开发好历史文化资源，传承人脉文脉，留住文化记忆。全力推进市奥体中心建设，推广全民健身，发展体育事业。加快养老服务产业发展步伐。推进健康萍乡建设和人口均衡发展，建立健全覆盖城乡居民的基本医疗卫生制度。依法按程序实施全面两孩政策。实施脱贫攻坚工程，全面落实精准扶贫工作，提前两年基本消除绝对贫困现象。继续推进政府应急管理工作。完善和落实安全生产责任和管理制度，强化预防治本，健全预警应急机制，坚决遏制重特大安全事故发生。推进平安建设、法治建设和廉政建设，完善化解矛盾机制。2016年工作安排

2016年是实施“十三五”规划的开局之年，是提前全面建成小康社会的关键之年。2016年全市经济社会发展的主要预期目标是：生产总值增长8%以上，财政总收入增长7%，全社会固定资产投资增长12%左右，社会消费品零售总额增长11%左右，城镇居民人均可支配收入和农村居民人均可支配收入分别增长9%和10%，居民消费价格总水平涨幅控制在2.5%左右，万元工业增加值能耗下降1.41%，城镇登记失业率控制在4%以内。为实现上述目标，主要抓好以下八个方面的工作：

突出创新发展，促进经济稳步增长

坚持创新驱动发展。深入实施创新驱动战略，推进大众创业、万众创新，增强发展动力和活力。以创建国家创新型试点城市为契机，突出科技创新企业主体地位，发挥政府引导职能作用，深入推进对外科技合作与交流，加快与国内外高等院校合作，逐步建立完善区域创新体系。全面推进科技入园入企，持续开展产学研合作。围绕重点产业集群，抓好科技协同创新和“智力”引进工作。加快国家级工业陶瓷工程技术研究中心萍乡分中心、粉末冶金院士工作站、国家花炮工程技术中心、国家电瓷工程技术中心、国家电瓷检验检测中心、国家海绵城市技术中心、市科技创新中心等科技公共服务平台建设。坚持科技创新企业主体地位，全面提升自主创新能力。

加快推进开放发展。加大招商引资力度，积极推行产业链招商、科技项目招商、以商招商、网络招商、驻点招商、小分队招商等多种招商方式，全年市县两级计划组织33场专题招商活动，突出引进战略投资者、大型央企和民企，大力发展总部经济，力争在引进世界500强企业上有新突破。持续实施“萍商回归”战略,充分发挥萍商资源优势，积极引导人才回乡、项目回归、资金回流、总部回迁。加大对外开放力度，加强新宜萍赣西区域发展。深入推进赣湘开放合作，全面贯彻落实赣湘开放合作试验区战略合作框架协议，建立联席会议机制，支持鼓励园区采取PPP等市场化模式推进一批重点工程。

扎实推进项目建设。把项目建设作为经济发展的强大动力，实施重大项目348个，总投资1086亿元。重点抓好华能安源电厂二期(2×1000MW)扩建工程、萍乡陶瓷产业基地西区扩区工程、江西紫金新能源汽车产业园项目、四方汽车科技有限公司年产40万台套汽车发动机零部件生产线建设项目、萍乡江能神雾煤炭分质梯级利用2×660MW超超临界发电示范项目等工业项目。以海绵城市、城区地下综合管廊、麻山生态新区、西气东输三线中段天然气管道工程等基础设施项目建设为契机，不断改善城乡环境。启动老煤校改造和保护，大力推进市奥体中心等民生工程项目。加快实施赣湘开放合作试验区的湘东区国家高新技术产业化基地和上栗县省级轻工产业基地等一批重大项目。不断创新融资模式。加大直接融资力度，用好PPP融资模式。加大力度落实战略性新兴产业(科技创新)引导资金信贷通平台、科技创新引导基金政策，完善财园信贷通、惠农信贷通等产业扶持政策，建立中小企业担保平台。力争以经营性收费方式，引入社会资金3.5亿元，参与生活垃圾、污泥干化焚烧发电项目建设。

坚持兴工强市，加速促进工业提质增效

着力抓传统产业转型发展。按照“一产一策、注重实效”的原则，依托创新引领，依靠联大联强，推动五大传统产业由粗放型向集约型转变，由资源优势向产业优势转化，实现传统产业新型化。着力培育壮大新兴产业。依托工业园区，以重点产业集群为基础，引导社会资源和资金向新兴产业、高科技企业聚集。重点发展节能环保陶瓷、高端电瓷、粉末冶金、先进装备制造、新能源汽车等产业，努力使新兴产业成为工业经济发展的重要力量。力争实现规模以上工业企业增加值455亿元，增长8%左右;主营业务收入1700亿元，增长5%;利税总额280亿元，增长6%。

着力抓好实体经济。推动供给侧结构性改革，帮助企业降低成本，包括降低制度性交易成本，企业税费负担、社会保险费、财务成本、电力价格、天然气价格、物流成本等，打出一套“组合拳”。实施企业成长工程，抓好“入规”企业培育，实施培育提升计划，放宽审批要求，优化办事流程，加强指导服务，壮大规上企业队伍，提升实体经济发展质量。强化实体企业扶持，继续深入开展市、县(区)领导挂点帮扶重点企业活动，支持萍安钢、萍矿、中煤科技等重点骨干企业转型发展。 加快推进工业园区发展。加大工业园区体制机制改革力度，探索市场化运作机制，以园区投资运营公司为主体，在投融资、土地开发等方面与市场接轨，实行规范的企业运作，力争在园区平台建设方面取得新突破。安源区、湘东区重点做好省级重点工业园创建工作，芦溪县积极推进省级生态园区建设工作，其他县区根据各自特色做好园区规划和建设。以萍乡经济技术开发区、丹江工业平台为试点，加快智慧工业园区建设，全力打造全省两化深度融合示范园区。

全力建设江南特色海绵城市，推进新型城镇化建设上台阶

全力推进海绵城市试点建设。按照统筹发挥自然生态功能和人工干预功能，有效控制雨水径流，实现自然积存、自然渗透、自然净化的城市发展方式，大力推进海绵城市试点建设，2016年预计投资26亿元。坚持PPP打包的原则重新组织系统化项目，科学合理确定田中湖片区项目包、玉湖片区项目包和老城区项目包内容。重点推进田中湖、玉湖治理，加快推进319国道、320国道、吴楚大道改造、市民广场建设，综合整治西门、万龙湾、中环南路、中环西路、山下路等积水点，加快鹅湖连通及调蓄工程、蚂蝗河合流制调蓄池建设等海绵城市项目建设。加快海绵城市PPP项目平台公司建设，综合运用多种融资工具吸引社会资本参与海绵城市试点建设，完成海绵城市监测平台和海绵城市展示馆建设。

着力抓好水生态文明城市试点建设。水生态文明城市试点建设计划投资13.44亿元，全市用水总量控制在8.65亿m3以内，万元工业增加值用水量降到60m3以下，农田灌溉水利用系数达到0.5，水功能区水质达标率达到85%以上。切实抓好灌区节水配套改造项目、山塘整治工程、长兴馆河污水干管和五丰河污水干管延长线建设等试点项目建设，努力打造“河畅、湖美、水清、岸绿、人水和谐”的水生态文明新萍乡。

大力抓好城市规划、建设和管理。科学规划城市，树立规划的权威性和指导性，稳步推进“多规合一”规划编制工作，全面开展城市设计，通过整合“经济社会发展规划、城乡总体规划、土地利用总体规划、生态环境保护规划”，实现市域管控“一张图”，着力谋划城市“成长坐标”。全面完成南正街旧城改造;重点推进中环北路、萍水南路延伸、萍安南大道延伸、登岸东路延伸等道路建设;着力推动荷叶塘棚户区改造、九形岭改造等老城区改造项目;继续建设田中湿地公园、聚龙公园、翠湖公园、鳌洲书院等;加快金融商务区建设，启动商会大厦、农商大厦、广电产业大厦、城市大厦等项目建设。努力解决交通拥堵，构建城区快速通道。推进城市防灾减灾和公共气象服务“两个体系”建设。加强城市管理，积极推进城市环卫工作市场化改革，推进城区路灯设施实行“合同能源”模式进行节能改造，大力实施城乡清洁工程，努力把萍乡建设成为干干净净、漂漂亮亮、井然有序、和谐宜居的城市。 全面推进交通电力基础建设。积极推动萍莲高速完成前期工作，争取早日开工建设。论证城际铁路网，积极对接长株潭城市群、推进新宜萍城市群建设。加快沪昆高速复线萍乡至醴陵段项目规划，积极争取列入国家、省高速公路网规划，并与湖南方面同步建设。确保国道319、320城区段和省道314樟排线(萍乡段)改造全面完工。完成公交城西水口综合客运站、开发区新能源公交车充电专用场站建设，加快推进公交北站、萍乡汽车客运中心站的前期工作。完成县乡道升级改造100公里，安全防护工程120公里，通自然村公路300公里。完成220KV大仓前输变电工程建设，提升多级电网供电保障能力。

着力抓好“三农”工作，促进农村经济发展

全力推进农业现代化。切实稳定粮食生产，全市粮食播面稳定在110万亩以上，总产确保在52万吨以上。着眼农产品产业链整体竞争力的提升和优势产业带的打造，促进农产品生产、加工与营销一体化发展。培育农业新型经营主体，加强农民合作社建设，大力发展家庭农场，力争实现省级以上龙头企业销售收入突破85亿元。大力调整农业产业结构，着力发展生态特色高效农业，推动水稻种植加工等传统产业转型升级，大力支持天涯种业发展壮大，全力打造在全国具有较强竞争力的育繁推一体化的现代种业集团。推动杂交水稻种业、油菜油茶、水产养殖等优势产业提质增效，实现从巩固面积向提升品质、延伸产业链转变;推动绿色生猪、花卉苗木、有机蔬菜、优质水果特色产业做大做强，进一步提升农业产业化水平。抓好芦溪国家级现代农业示范区和其他县区省级现代农业示范区规划建设的落实工作，努力建设好农产品精深加工产业园、五陂农业科技园等特色产业园。大力培育农业服务新业态，打造形式多样，特色鲜明的乡村旅游休闲产品，提高农业整体效益。 着力抓好农田水利等基础设施建设。加快推进高标准绿色生态农田，防洪抗旱减灾工程和农田灌溉“润田工程”建设，大力推进农业机械化建设，在省级以上农业示范园区创建一批全程机械化示范区，进一步改善农业生产条件，增强抵御自然灾害能力。完善建设用地收储制度，从严打击违法用地和违法建设现象，抓好耕地保护利用工作。大力推进各类水源工程项目，抓好寒山水库(中型)建设，启动碧湖潭、东源等中型水库建设前期工作，兴建20座小型水库引调提水工程。抓好萍水河防洪工程建设。加快中小河流治理、病险水库水闸除险加固、山洪沟治理等防洪薄弱环节建设。加大重点地区、重点流域水生态保护和水土保持力度。继续实施湘东区、芦溪县、上栗县、莲花县的小农水重点县建设项目。实施农村饮水巩固提升工程，提高农村自来水普及率、供水保证率和水质达标率。

抓好现代服务业，促进第三产业快速发展

加快旅游开发和文化产业发展。加快理顺旅游管理体制机构问题，落实兼职委员制度，抓紧成立全市旅游规划专业委员会。抓好武功山、安源景区两大产业集群建设，确保武功山游客服务中心投入使用。启用市旅游集散中心，加快推进安源景区游客中心、杨岐山普通寺重建、麻山月亮湾湿地公园、芦溪玉皇山景区、安源国家矿山公园、江西玉屏峰皇冠休闲旅游观光园等重点旅游项目，加强智慧旅游建设。确保完成武功山创建5A级景区资源评审，推进安源景区体制机制改革，深入发掘创建一批国家4A、3A级旅游景区。加快乡村旅游发展，围绕沪昆高速、319国道、320国道、湘东百里特色农业带、芦万武旅游公路等旅游通道，重点扶持和奖励一批乡村旅游项目，力争实现省5A级乡村旅游点零的突破。加快发展文化产业，制定出台文化产业发展政策措施，鼓励社会资本投资文化产业，着力培育文化企业，重点抓好安源文化博览园，萍乡中心书城、武功山文化产业园、莲花文化产业园、湘东彩印包装基地等重大项目和园区建设。加强红色文化教育和培训。加快发展文化新兴业态，促进文化与旅游、互联网、科技、农业休闲、体育等产业融合发展，把文化产业打造成转型升级的新引擎和新的支柱产业。

促进金融业等稳定发展。继续抓好中小企业信用体系建设，推进各县区信用体系建设优化升级。加强县区政银企合作工作机制，不断推进企业上市工作。进一步扩大直接融资，加强金融机构建设，实现村镇银行在各县区全覆盖，加快现有金融机构向县区延伸。做好重点企业金融风险早期识别试点等金融创新工作，创新开展金融扶贫，防范化解金融风险，坚决守住不发生系统性和区域性金融风险的底线。化解房地产库存压力，通过加快农民工市民化，扩大有效需求，稳定房地产市场，深化住房制度改革，进一步优化住房公积金使用政策，建立购租并举的住房制度，发展住房租赁市场，鼓励发展以住房租赁为主营业务的专业化企业，鼓励房地产开发企业顺应市场规律调整营销策略，促进房地产业兼并重组，提高产业集中度。 加快实施“互联网+”等行动。通过制定科学规划，推广普及电子商务应用，发展移动电子商务。积极实施“互联网+”示范带动工程，加快产业基地建设，搭建互联网平台体系。大力促进电商发展，坚持以规划引导发展，编制完成萍乡市电子商务发展规划，着力打造具有萍乡特色的电子商务产业带。大力发展“互联网+文化”产业，搭建公共技术服务平台，重点发展动漫创意企业，打造动漫创意集聚区。

推进绿色崛起，不断改善生态环境

大力发展绿色经济。将工业节能与资源综合利用全面融入工业化、城镇化进程，着力推进绿色发展、循环发展、低碳发展，大力发展绿色制造业、节能环保产业、节能服务产业、循环经济产业。推动建立以企业为主体、产学研相结合的节能技术创新体系，推动组建以市场为导向、多种形式相结合的节能技术与装备产业联盟。深入贯彻实施新《环保法》和《大气污染防治法》，继续加大总量减排和污染防治力度，严格环境执法，依法依规，严厉打击环境违法行为。完善治污基础设施，全市所有工业园区污水处理厂今年必须建成投入营运，加大城市污水管网建设改造力度。 巩固提升生态优势。加快推进生态文明先行示范区建设步伐，加强造林绿化建设，全面完成人工造林6.3万亩，森林抚育29.3万亩。继续全面封山育林，着力加强林业资源保护，提高森林防火综合防控能力。启动实施引调提水工程、农村生活垃圾整治等项目建设。

保障和改善民生，促进社会和谐

不断提升社会保障水平。着力抓好民生工程，完成就业和创业、社会保障、社会救助、医疗保障等八个方面的民生工程指标。以国家鼓励“大众创业、万众创新”为契机，以创业带动就业。新增城镇就业2.61万人，新增转移农村劳动力1.67万人。提高社会保险水平，城镇基本养老保险参保缴费36.2万人，基本养老保险基金征缴总额13.47亿元，被征地农民参加养老保险4万人。提高医疗保障水平，城乡居民基本医疗保险参保缴费111.1万人。加大保障性住房建设力度，建设保障性安居工程11035套，其中城市棚户区7975户、其他类棚户区2267户、公租房793套。大力推行新建、以购代建、以租代建以及货币化安置等相结合的组合模式，完成保障性安居工程建设任务。进一步提高社会救助水平，稳步提高城乡低保、农村“五保”、城镇“三无”、精简退职人员等困难群众保障标准。统筹推进养老服务体系建设，全面推进市社会福利中心项目和湘东、芦溪、上栗3个县级福利中心工程建设，重建或改造提升20所乡镇敬老院，支持社会力量发展养老服务，推动麓林湖养生养老公馆等项目建设。

全力抓好脱贫攻坚工作。科学制定脱贫攻坚“时间表”和“路线图”。围绕既定目标，层层传导压力，确保责任落实。逐层细化脱贫“时间表”。认真落实《关于调整2015-2018年脱贫指导性计划的通知》文件要求。将脱贫计划任务按年度层层分解落实到县区、到乡镇、到村、到户、到人，确保2018年实现脱贫目标。按照市县有规划、乡村有计划、农户有卡册的要求，逐层科学制定脱贫“路线图”。市、县区两级要制定精准脱贫规划，提出时限要求，明确责任主体，统筹工作布局，扎实推进扶贫规划有效实施。全面落实精准扶贫工作，实施“一对一、点到点”精准帮扶。

大力发展社会事业。努力提升义务教育均衡发展水平。全面推进素质教育，健全从学前教育到义务教育、高中、中职教育以及大学教育的资助体系，扩大资助覆盖面。争取建设完成玉湖学校一期工程、市级教育公共服务平台。加大中小学校舍危房改造、教育信息化项目建设。不断提升现代职业教育。进一步落实贫困学生和农村中职学生免除学杂费政策，为全市1105名农村建档立卡贫困户中在读高中生免除学杂费，落实边远艰苦地区、特岗教师人才计划。继续完善全民健身服务体系，加快竞技体育改革步伐。全力推进县级公立医院综合改革，启动城市公立医院改革，加快建立和完善现代医院管理制度，积极推进分级诊疗制度的实施。加快推进社会办医，落实相关政策待遇。建立健全全民医保体系，完善贫困家庭部分大病免费救治长效机制，深化医保支付方式改革，全面建立大病保险、疾病应急救助等制度，健全药品保障供应机制等。依法有序推进两孩政策落地。大力实施乡村亮化工程。加强人防工程建设，提升城市综合功能。加快气象现代化建设，提高气象灾害防御能力。 努力维护社会安全稳定。落实新《安全生产法》和安全生产“党政同责、一岗双责、失职追责”，强化“底线”思维和“红线”意识，提升全市安全生产总体水平。推进烟花爆竹、煤矿、非煤矿山、危险化学品的安全生产条件和安全管理“双达标、双规范”，全面提升企业安全管理水平。用法治思维解决信访问题，规范信访秩序。推进社会治安防控体系建设，净化网络空间，坚决依法打击“瞎闹”，对多发性侵财犯罪、“黄赌毒”以及损害经济发展环境的违法犯罪行为，继续保持严打高压态势，进一步提高群众安全感满意度。

坚持依法行政，不断加强政府自身建设

依法履行政府职能。坚持法定职责必须为、法无授权不可为，科学确定行政机关职能和事权，推进政府机构、职能、权限、程序、责任法定化。进一步推进政企分开、政资分开、政事分开、政府与市场中介组织分开，厘清政府与企业、市场、社会的界限。健全政务公开机制，切实保障人民群众的知情权、参与权、表态权和监督权。接受市人大常委会的依法监督和市政协的民主监督，认真办理人大代表建议、议案和政协委员提案。
